# *Aphanizomenon flos-aquae*: A Biorefinery for Health and Energy—Unleashing Phycocyanin’s Power and Biogas Potential

**DOI:** 10.3390/md23060225

**Published:** 2025-05-24

**Authors:** Pilar Águila-Carricondo, Raquel García-García, Juan Pablo de la Roche, Pedro Luis Galán, Luis Fernando Bautista, Juan J. Espada, Gemma Vicente

**Affiliations:** 1 Department of Chemical, Energy and Mechanical Technology, Universidad Rey Juan Carlos, C/Tulipán s/n, 28933 Móstoles, Spain; raquelg2ac@gmail.com (R.G.-G.); juanjose.espada@urjc.es (J.J.E.); 2 Microalgae Solutions S.L., Factoría Industrial de Vicálvaro, Nave 5, 28052 Madrid, Spain; jproche@microalgaesolutions.com (J.P.d.l.R.);; 3 Department of Chemical and Environmental Technology, Universidad Rey Juan Carlos, C/Tulipán s/n, 28933 Móstoles, Spain; fernando.bautista@urjc.es; 4 Instituto de Tecnologías para la Sostenibilidad, Universidad Rey Juan Carlos, C/Tulipán s/n, 28933 Móstoles, Spain

**Keywords:** *Aphanizomenon flos-aquae*, cyanobacteria, antioxidant, U-118 MG brain glioma cells, HCC1806 breast cancer cells, biomethane, biogas, biorefinery, LCA

## Abstract

This study presents a biorefinery approach for *Aphanizomenon flos-aquae*, demonstrating its potential as a dual source for phycocyanin and biogas. The antioxidant capacity of the extract was evaluated using the ABTS^•+^ assay, while flow cytometry determined its cytotoxic effects on breast cancer (HCC1806) and brain glioma (U-118 MG) cell lines, comparing pure C-phycocyanin to the non-purified extract. The non-purified extract scavenged 77% of ABTS^•+^ radicals at 2.4 mg/mL, compared to 22% for pure C-phycocyanin. In U-118 MG cells, pure C-phycocyanin accounted for 55.5% of the 29.9 ± 6.1% total mortality observed with the non-purified extract at 0.75 mg/mL. HCC1806 cytotoxicity (80.9 ± 5.1% at 1 mg/mL) was attributed to synergistic effects of other extract components. The spent biomass was valorized through anaerobic digestion for biogas production, enhancing process sustainability. At a 2:1 inoculum-to-substrate ratio, the anaerobic digestion of the spent biomass yielded 447 ± 18 mL CH_4_/gVS, significantly higher than the 351 ± 19 mL CH_4_/gVS from the initial biomass. LCA estimated the environmental impacts of the *A. flos-aquae* biorefinery for phycocyanin production, targeting the cosmetic, food, and nutraceutical sectors, and highlighting the benefits of spent biomass valorization to produce biogas within a circular economy framework. This integrated approach demonstrates the potential of *A. flos-aquae* for the sustainable production of high-value compounds and renewable energy.

## 1. Introduction

Cyanobacteria are a morphologically diverse group of oxygenic photoautotrophic and Gram-negative prokaryotes, comprising unicellular, colonial, and filamentous forms. Certain filamentous strains exhibit a multicellular lifestyle, with intercellular communication and functional cell differentiation. They are widely distributed in terrestrial and aquatic environments, predominantly found in freshwater and marine ecosystems [1]. Nowadays, Cyanobacteria have become increasingly important, primarily for their potential biotechnological applications in the food and pharmaceutical industries, as well as in biofuel production and wastewater treatment. They constitute an incredibly valuable bioresource due to their ability to photosynthesize and produce a wide range of high-value metabolites, including pigments, vitamins, lipids, carbohydrates, and proteins. Their fast growth, ability to thrive in diverse environments, and potential for genetic engineering make them a promising resource for sustainable bioproduct development [2].

One of the primary applications of Cyanobacteria today is in the food and supplement industries, where their high nutritional value and protein content make them essential ingredients. These microorganisms, particularly species like *Limnospira*/*Arthrospira* sp. and *Nostoc* sp., are rich in essential amino acids, vitamins, and antioxidants, making them popular as sustainable dietary supplements. *Nostoc*, for example, is consumed in many Asian cuisines for its nutritional benefits and is valued for its nitrogen-fixing ability [3]. Their capability to provide a natural and sustainable source of protein has also led to their use in functional foods and nutritional products. In addition, Cyanobacteria are recognized for their impressive nutritional profile, which contributes to their growing presence in markets focused on health and wellness [4]. Currently, *Limnospira*/*Arthrospira* sp. dominates these markets, but other Cyanobacteria, such as *A. flos-aquae*, possess unexplored bioengineering prospects [5].

The unicellular cyanobacterium *A. flos-aquae* grows in brackish and freshwater and is commonly known as a blue-green alga [6]. The rapid proliferation of this cyanobacterium can cause excessive surface water blooms [7]. For instance, it naturally proliferates in large quantities in Upper Klamath Lake (Oregon, USA) [8] and Curonian Lagoon (Lithuania and Russia) [9]. The removal of wild cyanobacterial blooms has been proposed as a key management measure, as some cyanobacterial species produce toxins that can negatively impact both health and the environment. In the case of *A. flos-aquae*, while some studies have reported the presence of toxin-producing strains within the *Aphanizomenon* genus, including *A. flos-aquae* [10,11], other analyses did not detect toxin production in bloom samples or in culture isolates from the Curonian Lagoon [12,13]. In fact, it is considered a highly nutritious food source and is widely consumed as a nutritional supplement for its health benefits [14], having been approved for human consumption by both the U.S. Food and Drug Administration (FDA) and the European Food and Safety Authorities (EFSA) [5].

Like other Cyanobacteria, *A. flos-aquae* contains high levels of phycobiliproteins, such as C-phycocyanin (up to 10% by dry weight), giving it its characteristic blue color. Phycocyanin, with its potent antioxidant, anti-inflammatory, and antitumor properties, has garnered increasing attention for its potential therapeutic benefits [15,16]. Its ability to inhibit cancer cell growth and reduce inflammation makes it a promising, natural, and sustainable compound for cancer treatment. While several studies have reported the bioactivities of C-phycocyanin-rich extracts for different species, as highlighted in interesting reviews [17,18,19], there is limited research specifically on the antioxidant and antitumor effects of phycocyanin extract from *A. flos-aquae*. Benedetti et al. [8,20] were the first to demonstrate the antioxidant activity of a phycocyanin extract from this cyanobacterium. Regarding the cytotoxicity assays, the *A. flos-aquae* ethanolic extracts inhibit both HL-60 and MV4-11 leukemic cell lines [21] Only Syrpas and co-workers [7] jointly observed in vitro antioxidant capacity in the Folin−Ciocalteu and ABTS^•+^ assays, as well as a cytotoxic effect against C6 glioma cells (a rat glioma cell line) for C-phycocyanin extracts of wild *A. flos-aquae*. While the potential of phycocyanin-rich extracts in cancer research has been explored, and various studies have investigated their bioactivities against different cancer cell lines, this study presents a new investigation into the in vitro cytotoxic effects of a phycocyanin-rich extract from *A. flos-aquae* (not previously tested) on two human cancer cell lines: the U-118 MG glioblastoma and the HCC1806 breast cancer cell lines. This is particularly relevant as U-118 MG represents glioblastoma, the most aggressive, prevalent, and lethal type of brain cancer [22], while HCC1806 is representative of a common form of breast cancer [23]. Therefore, this research contributes novel data regarding the potential of this specific *A. flos-aquae* extract against these significant human tumor models, opening new lines of research in cancer therapy studies.

Once the phycocyanin-rich extracts are obtained, the residual cyanobacterial biomass from *A. flos-aquae* can be used in the production of bioproducts or bioenergy within a zero-waste biorefinery approach. Anaerobic digestion stands out as one of the most promising and commercially viable technologies in algal and cyanobacterial biorefinery design, particularly within the context of Europe’s ongoing sustainability initiatives. With the European Green Deal aiming for carbon neutrality by 2050, anaerobic digestion offers an effective method for converting cyanobacterial waste biomass into renewable energy, helping to meet the EU’s ambitious climate targets and contributing to the development of a low-carbon economy. Anaerobic digestion is a multi-stage microbial process comprising hydrolysis, acidogenesis, acetogenesis, and methanogenesis, whose efficiency can be affected by the biochemical composition of the substrate. When using cyanobacterial biomass, challenges such as low biodegradability, unfavorable C/N ratios, or the presence of inhibitory compounds may arise and should be considered in process design. Additionally, it involves the mineralization of organic nitrogen and phosphorus in the residual biomass, with the resulting digestate serving as a valuable soil amendment and fertilizer [24]. In previous studies, we tested spent biomass from different microalgal and cyanobacterial species: *Microchloropsis gaditana*—formerly *Nannochloropsis gaditana*—(*Eustigmatophyceae*), *Isochrysis galbana* (*Coccolithophyceae*), *Limnospira*/*Arthrospira* sp. (Cyanobacteria), and *Dunaliella salina* (Chlorophyta), obtained through various bioproduct extraction processes, as feedstock for anaerobic digestion. These studies yielded promising biogas results [25,26,27]. However, to date, there have been no reports in the literature on the anaerobic digestion of both intact and spent biomass derived from bioproduct extraction processes from *A. flos-aquae*.

In the present work, we investigate a multi-product biorefinery based on the cyanobacterium *A. flos-aquae*, aimed at jointly obtaining the following: (i) phycocyanin rich-extracts with antioxidant properties and a cytotoxic effect on cancerous cell lines, which can be further explored for the applications in the cosmetic, nutraceutical, or pharmaceutic industry, and (ii) biogas with a relevant yield and quality for the bioenergy industry, all within the context of a circular economy. In this context, assessing the sustainability of the proposed biorefinery is essential, and tools such as life cycle assessment (LCA) play a key role. We incorporated LCA to comprehensively understand the environmental impacts at each stage of the biorefinery, identifying key areas for improving efficiency and reducing negative impacts. This evaluation is crucial to ensure that our multi-product biorefinery is not only technologically feasible but also environmentally responsible, supporting the transition towards a circular and sustainable economy.

## 2. Results

### 2.1. Aqueous Extraction

*A. flos-aquae* is a protein-rich cyanobacterium, since the total protein content was 65.7 ± 3.2% (Table 1). Carbohydrates were the second group of biomolecules in order of abundance in this species (19.0 ± 0.3%), while lipids were the least abundant biomolecule (5.9 ± 0.1%). After aqueous extraction and filtration, the initial biomass was distributed as follows: 43.2 ± 0.3% was extracted, and 26.9 ± 0.1% remained in the spent biomass. The aqueous extraction recovered higher proportions of proteins and carbohydrates (46.7 ± 2.8%, and 45.6 ± 2.8%, respectively). *A. flos-aquae* spent biomass contained 30.5 ± 0.6% of proteins and a small amount of carbohydrates (13.0 ± 0.8%) and lipids (15.9 ± 2.5%). Biochemical characterization pointed out that the aqueous extract and spent biomass were rich in proteins. Carbohydrates were more abundant in the extract than in the spent biomass. The low lipid recovery results in both the aqueous extract and the spent biomass are attributed to the low lipid content in the initial biomass.

The blue pigment, phycocyanin, yielded 41.9 ± 0.6 mg/g. Syrpas et al. [2] obtained similar phycocyanin extracts from *A. flos-aquae* (49.2 ± 0.2 mg/g) using a methodology that combined freeze–thaw cycles and ultrasounds. The phycocyanin yield represented 13.1% and 9.7% of the total proteins and dry biomass quantified in the extract, respectively. The *A. flos-aquae* aqueous extracts produced in this work are of interest to industries such as food, cosmetics or pharmaceutics due to their high protein content and, on the other hand, their high phycocyanin content, which is known for its reported antioxidant and anticancer properties [5].

#### 2.1.1. *A. flos-aquae* Extract Exhibits Antioxidant Properties

**Figure 1:**
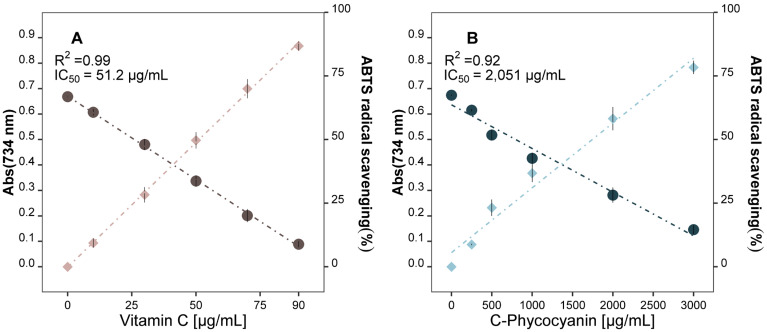
Antioxidant capacity of (**A**) vitamin C, and (**B**) C-phycocyanin pure standards, measured by ABTS assay. Absorbance at 734 nm (circles); ABTS radical scavenging % (rhombuses).

We studied the antioxidant power of the *A. flos-aquae* aqueous extracts (non-purified) as their capacity to scavenge ABTS^•+^ radicals using two standards: L(+)-ascorbic acid (vitamin C), and pure C-phycocyanin (Figure 1).

The results obtained from the ABTS^•+^ assays showed that both standards scavenged ABTS^•+^ radicals in a dose-dependent manner: as the compound concentration increased, the scavenging potential also increased. Vitamin C released higher antioxidant power than C-phycocyanin, with IC_50_ values of 51.2 µg/mL and 2051 µg/mL, respectively.

*A. flos-aquae* aqueous extract was assessed in terms of antioxidant capacity and expressed as vitamin C equivalents (VCEAC) and C-phycocyanin equivalents (C-PC Eq). The results are shown in Table 2.

*A. flos-aquae* aqueous extracts released 77.0 ± 0.7% of ABTS^•+^ radical scavenging at a concentration of 2.4 mg/mL. Other non-purified cyanobacterial aqueous extracts have been studied to assess their potential to scavenge ABTS^•+^ radicals. Aqueous extracts from *Limnospira platensis* (formerly *Arthrospira platensis*) were obtained by freeze–thawing and ultrasounds (similar methodology to the one used in our work), obtaining a lower ABTS^•+^ scavenging potential (60% of scavenging activity at 2.5 mg/mL [28]). *Anabaena cylindrica* aqueous extracts were produced using thermomixing [29]. These extracts showed also lower values (40–50% using 2.5 mg/mL) compared to the cyanobacterial extracts presented in this work. Differences in antioxidant power may be explained by the varying procedures and cyanobacterial species used. Extraction techniques should break down cell walls allowing phycocyanin to be extracted, while also affecting the diffusion of the solvent. Moreover, phycocyanin content varies not only with the cyanobacterial species but also with cultivation conditions [30].

On the other hand, many efforts have been made in performing procedures to purify cyanobacterial aqueous extracts to obtain crude or purified C-phycocyanin, especially from *L. platensis* [31,32,33,34] as it is considered to possess higher bioactivity than non-purified extracts. However, while some of the purified or partially purified phycocyanin in those works released higher ABTS^•+^ scavenging potential than the aqueous extracts of *A. flos-aquae* extracts in the present research [32,33], others released lower ABTS^•+^ scavenging power [34], showing that there are multiple factors affecting the antioxidant ability of aqueous extracts from Cyanobacteria and suggesting that C-phycocyanin is not always the major responsible for the bioactivity. In this regard, Macario et al. [29] suggested that the bioactivity of rich C-phycocyanin extracts should not be associated with the blue pigment content, as they found that non-purified extracts were more bioactive than purified ones. The *A. flos-aquae* non-purified extracts produced in this work released an antioxidant power of 235 ± 2.1 µg VCEAC/mL and 9971 ± 90 µg C-PC Eq/mL (Table 2), showing that C-PC is not as effective as vitamin C to scavenge ABTS^•+^ radicals. To assess the extent to which the antioxidant power of the produced aqueous extract was related to its phycocyanin content, we calculated the ABTS^•+^ radical scavenging activity of the corresponding C-phycocyanin quantified in the extracts. The results pointed out that the pigment was responsible for 22% of the whole antioxidant power of the aqueous *A. flos-aquae* extracts, confirming the results obtained by Macario et al. [29]. These results highlight the high antioxidant potential of non-purified extracts. Other research [35] studied the antioxidant power of different pigments isolated from the cyanobacterium *L. platensis*, and they found that although phycocyanin exhibited antioxidant power, it was the pigment with the least bioactivity in terms of antioxidant capacity.

#### 2.1.2. *A. flos-aquae* Extract Exhibits Cytotoxicity Against U-118 MG and HCC1806 Tumoral Cell Lines

**Figure 2:**
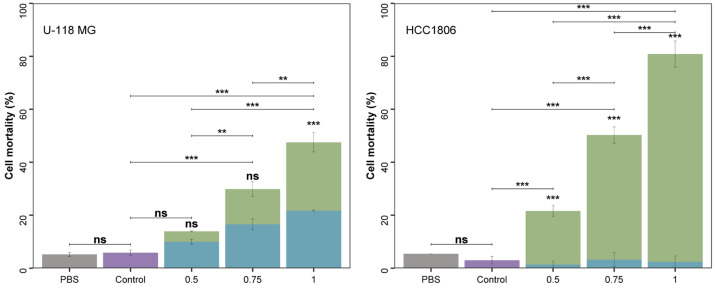
Effects of *A. flos-aquae* extract (mg/mL, dry basis) in green and corresponding pure C-PC in the *A. flos-aquae* extract in blue on the viability of U-118 MG cells and HCC1806 cells after 48 h, measured with flow cytometry. Control experiments (purple) and PBS experiments (gray). ns: no significant differences; **: 0.01–0.001; and ***: 0.001–0. Replicates = 3.

Water extracts of *A. flos-aquae* were tested in in vitro cytotoxicity assays in two cell lines: human brain glioma cells (U-118 MG) and breast cancer cells (HCC1806), as shown in Figure 2.

The cytotoxicity assays revealed that the aqueous extract from *A. flos-aquae* can inhibit the proliferation of brain glioma cells (U-118 MG) and breast cancer cells (HCC1806) in a dose-dependent manner when concentrations of 0.5, 0.75, and 1 mg/mL were tested. The cytotoxic effect was higher for breast cancer HCC1806 than for brain glioma U-118 MG cells. PBS solution did not influence cell mortality in any case as no significant differences were found in cell mortality of control and PBS experiments.

For brain glioma U-118 MG cells, cell mortality reached 47.5 ± 6.7% with 1 mg/mL of the extract, showing significant differences with the other concentrations tested. *A. flos-aquae* extracts at 0.75 mg/mL and 0.5 mg/mL produced a cell mortality of 29.9 ± 6.1% and 13.9 ± 0.7%, respectively, with significant differences among them. The cytotoxic results offered by *A. flos-aquae* extract concentrations were significantly different from the control, except for 0.5 mg/mL. *A. flos-aquae* was tested previously against rat glioma cells (C6). Incubation at 72 h produced a mortality rate of more than 90% with an extract concentration of 0.1 mg/mL [7]. Other Cyanobacteria, like *Choococus minutus*, *Anagnostidinema carotinosum* (formerly *Gleiterinema carotinosum*), *Nostoc linckia*, and *Nostoc oryzae* (formerly *Anabaena oryzae*), were assayed as cytotoxic agents against this cell line, confirming higher mortality rates [36] than those of the present study with the human cell line U-118 MG. This finding suggests that cell line animal in vitro testing presents limitations that often overestimate the results compared to human cell lines, in this case U-118 MG, and reveals the importance of performing experiments in real human cell lines, except in animal ones. Moreover, cyanobacterial species selection is crucial as they differ in the composition of bioactive molecules.

**Figure 3:**
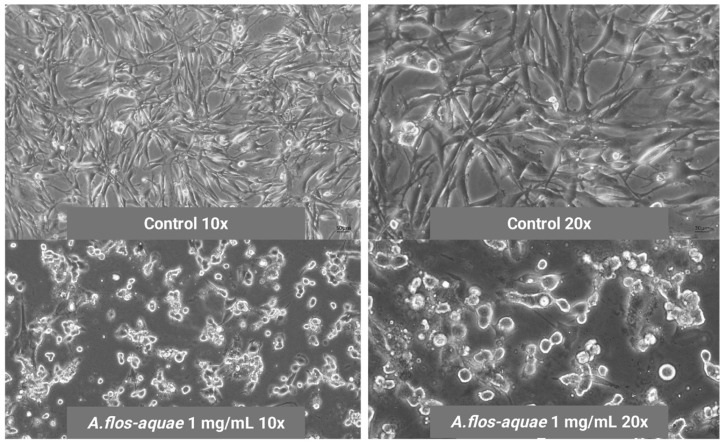
Mortality signs of Brain Glioma U-118 MG cells when incubating with 1 mg/mL of *A. flos-aquae* aqueous extract compared to healthy control U-118 MG cells without *A. flos-aquae* extract (control).

Morphological signs of cell mortality were observed in microscopy images at different magnifications (Figure 3). The treatment with *A. flos-aquae* extract at 1 mg/mL produced a significant reduction of U-118 MG cell volume and apoptotic bodies appeared around the cells.

The breast cancer HCC1806 cell line was more vulnerable than U-118 MG cell line, reaching a cell mortality of 80.9 ± 5.1% with 1 mg/mL, differing significantly with all the experimental treatments. The medium concentration (0.75 mg/mL) yielded a mortality rate of 50.3 ± 3.3%, a statistically different result compared to that obtained with the minimum concentration of 0.5 mg/mL, which produced a cell mortality of 21.6 ± 2.2%. In another study, *A. flos-aquae* extract was incubated with T47D cells (human breast adenocarcinoma) [9], obtaining a high mortality rate of 97% with only 0.2 mg biomass per mL. This difference may be due again to differences in cell line due to their different nature and physiology. HCC1806 cells are more proliferative than T47D, based on the results of the proliferation marker Ki67, investigated in a previous study [37].

**Figure 4:**
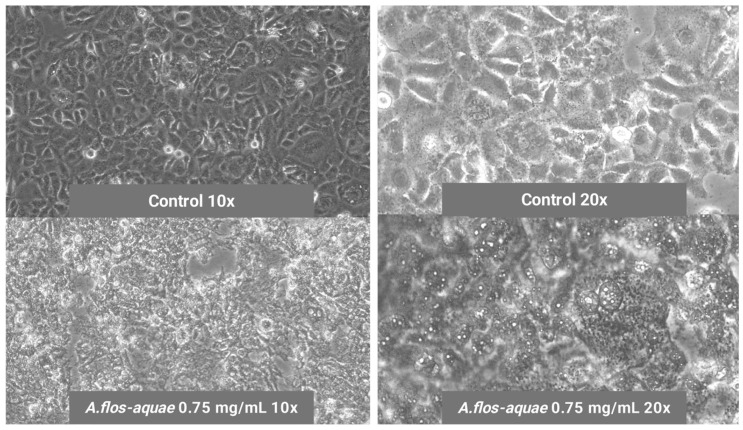
Mortality signs of breast cancer HCC1806 cells when incubating with 0.75 mg/mL of *A. flos-aquae* aqueous extract compared to healthy control HCC1806 cells without *A. flos-aquae* extract (control).

As in the case of brain glioma U-118 MG cells, microscope images revealed changes in the morphological aspect of HCC1806 cells when incubated with *A. flos-aquae* extracts, showing mortality signs such as abnormal morphology and apoptotic accumulated bodies (Figure 4).

The effect of C-phycocyanin present in *A. flos-aquae* extracts (using a commercial standard) was assessed to investigate the extent to which this blue pigment was responsible for the antitumoral effect (Figure 2).

The results indicated that C-phycocyanin had a considerable effect on cell mortality compared to the whole extract in the U-118 MG cell line. At 0.05 mg C-PC/mL (quantity of C-PC contained in 0.5 mg/mL of *A. flos-aquae* extract), and 0.07 mg C-PC/mL (quantity of C-PC contained in 0.75 mg/mL of *A. flos-aquae* extract), cell mortality reached 71.7% and 55.5% (regarding the total mortality produced by the whole extract). However, results at 0.05 mg C-PC/mL did not differ from the control experiment. On the other hand, cell mortality caused by 0.07 mg C-PC/mL was significantly different from control, and did not exhibit differences with the *A. flos-aquae* extract at 0.75 mg/mL. This result suggests that the antitumoral effect may be attributed to the C-PC present in the *A. flos-aquae* extracts. However, at 0.09 mg C-PC/mL (corresponding to 1 mg/mL of *A. flos-aquae* extract), C-PC was responsible for 47.8% of the total cell mortality caused by the whole extract, displaying significant differences compared to the entire extract. This means that C-phycocyanin is primarily responsible for the antitumoral effect of the extracts, but other compounds may enhance the cytotoxicity of the extracts over U-118 MG cells, especially at higher concentrations (1 mg/mL).

In the case of the HCC1806 cell line, it may be less vulnerable to C-PC, as the contribution of this pigment to total cell mortality was in the range of 2.9% to 6.4% at the tested concentrations, showing significant differences with the whole extract. The results point out that the major responsible for the in vitro antitumoral capacity in this cell line was not C-PC, unlike for the U-118 MG cell line, but other compounds present in the *A. flos-aquae* extract. This result is in line with previous research that found a non-purified extract of a cyanobacterium (*Anabaena cylindrica*) to be more cytotoxic against human melanoma MNT-1, cells compared to a purified extract with purer C-PC [29].

These antitumoral results should be considered as a preliminary approach to understanding the bioactivity of complex non-purified extracts compared to purified ones. However, it should be mentioned that for pharmaceutical or medical treatments, extracts must be purified according to regulations. Moreover, further studies should explore the effect of these extracts on the corresponding healthy cell lines and scale up the experiments to corroborate the efficacy of the *A. flos-aquae* aqueous extracts.

### 2.2. Production of Biogas from Spent Biomass

**Figure 5:**
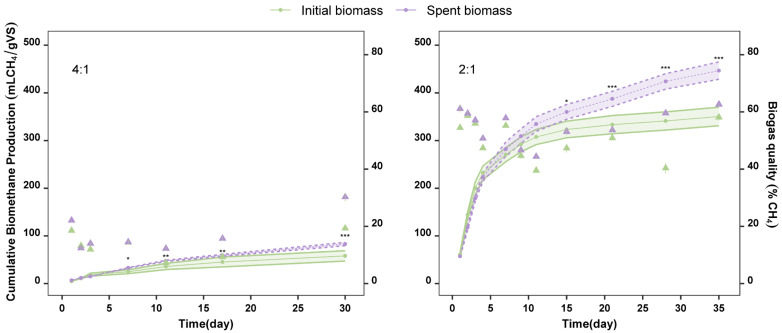
Cumulative biomethane production (lines) and biogas quality (triangles) of initial biomass (green) and spent biomass (purple) of *A. flos-aquae* using inoculum to substrate ratios of 4:1 and 2:1. Initial biomass vs. spent biomass *p*-value: 0–0.001: ***; 0.001–0.01: **; 0.01–0.05: *.

Spent biomass obtained after the water extraction of *A. flos-aquae* was assessed for its potential to produce biogas and compared to biomethane yields produced by the intact *A. flos-aquae* biomass. Control experiments were conducted with the sludge without substrate, and biomethane production values were subtracted. Two inoculums-to-substrate ratios were studied: 4:1 and 2:1. The cumulative biomethane kinetic curves are depicted in Figure 5.

**Figure A1:**
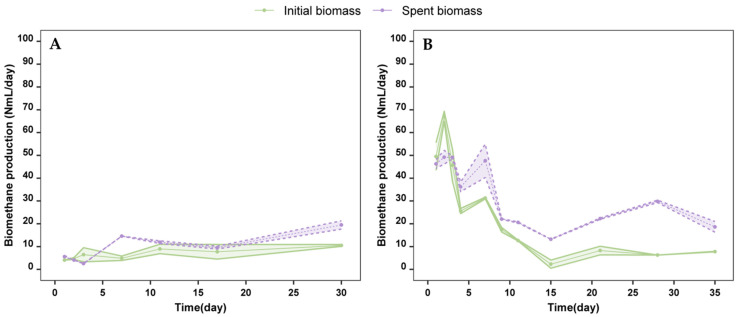
Biomethane production per day using initial biomass (green) and spent biomass (purple) of *A. flos-aquae* using inoculum-to-substrate ratios of 4:1 (**A**) and 2:1 (**B**). Normal conditions (T = 273.15 K, P= 101,325 kPa).

Anaerobic digestion performed at 4:1 yielded low biomethane production. Although the biomethane yield increased in both biomasses over time (Figure A1 in the Appendix A), it remained too low by day 30, which was the final day of the study at this ratio. On that day, the values were slightly higher for the spent biomass substrate (83 ± 4 mL CH_4_/gVS) compared to the initial biomass substrate (58 ± 11 mL CH_4_/gVS), (Figure 5). Differences in biomethane production due to the type of substrate were significant (*p*-value < 0.05) from day 7. From day 11 to day 30, the difference in the biomethane yields between the spent biomass and the initial biomass became even more pronounced (*p*-value < 0.001), reaching the highest significance on day 30.

Biomethane production was significantly higher at an inoculum-to-substrate ratio of 2:1 compared to 4:1. These findings are consistent with other research that also tested the influence of the ratio of inoculum to microalgae [38]. Spent biomass yielded 447 ± 18 mL CH_4_/gVS, and initial biomass 351 ± 19 mL CH_4_/gVS on day 35 (end of the study) using a 2:1 inoculum-to-substrate ratio. The experiments at this ratio also released higher cumulative biomethane yields using the spent biomass from day 7 to day 30. Differences in the type of substrate used became noticeable on day 15 (*p*-value < 0.05). From day 21 to day 35, the biomethane yield from the spent biomass substrate increased significantly over time, showing marked differences (*p*-value < 0.001) compared to the initial biomass substrate, whose yields did not increase as much over time.

To our knowledge, this is the first study to assess intact and spent *A. flos-aquae* biomass in anaerobic digestion. In our study, the initial biomass of *A. flos-aquae* offered higher biomethane yields (351 ± 19 mL CH_4_/gVS) at an inoculum-to-substrate ratio of 2:1 compared to *Umezakia ovalisporum*—formerly *Aphanizomenon ovalisporum*—(Cyanobacteria) (287.7 mL CH_4_/gVS), a species previously evaluated for its biomethane production potential [39]. These values fall within the intermediate range of biomethane yields reported for other cyanobacterial species, including *Synechocystis* sp., *Dolichospermum planctonicum* (formerly *Anabaena planctonica*), and *Borzia trilocularis*, which have been used as substrates and yielded between 255 and 380 mL CH_4_/gVS under comparable conditions. Similarly, our results fall within the broad range observed for *L. platensis*, the most extensively studied cyanobacterium in biogas production, with reported biomethane yields varying from 181 to 481 mL CH_4_/gVS [40,41,42].

Cyanobacterial-biorefinery approaches using species other than *A. flos-aquae* have been previously studied, including the extraction of phycobiliproteins and the assessment of the biomethane production of the initial biomass compared to the spent biomass (after extraction). Bellver et al. [43] pointed out a reduction in the biomethane production potential of the spent biomass of *Synechococcus* sp. compared to the yields obtained with the initial biomass substrate, a trend opposite to that observed in our study. However, a study on the anaerobic digestion of the initial biomass of *L. platensis* found lower biomethane yields than those of its spent biomass after protein extraction, like the trend observed in our study [41]. Similarly, lipid-extracted biomass from *L. platensis* offered higher biomethane values than those offered by the initial biomass [44], as observed in our study with the spent biomass. These differences could be attributed to variations in cell membrane composition among cyanobacterial species or the different extraction methods used. Both factors highly influence the availability of soluble organic matter as well as the biodegradability of the compounds. From day 3 onward, the spent biomass offered higher daily biomethane production than the initial biomass (Figure A1 in Appendix A). In this regard, methanogenic bacteria find it easier to access biomolecules and convert organic compounds into CO_2_ and CH_4_ when using spent biomass as substrate rather than initial biomass.

As shown in Table 3, an increase of 2.4 and 1.9 in COD was observed employing initial and spent biomass in a 4:1 experiment. This suggests that anaerobic digestion did not proceed appropriately in this case, likely due to the transformation of organic matter to organic acids, which would increase the soluble COD [45]. Indeed, the observed pH changes from 6.3 ± 2 × 10^−2^ to 5.3 ± 2 × 10^−2^ and from 6.4 ± 0 to 5.3 ± 2 × 10^−2^ for the initial and spent biomass, respectively (Table A1 in the Appendix A), indicating medium acidification, likely due to the accumulation of organic acids. Conversely, COD changes from day 1 to the final day in the 2:1 experiment indicated that anaerobic digestion was properly carried out, as evidenced by the 6.3 and 1.8-fold decrease when using the initial and spent biomass, respectively. In this case, pH changes indicated more basic conditions: from 6.9 ± 3 × 10^−2^ to 7.9 ± 5 × 10^−2^ and from 7.0 ± 4 × 10^−2^ to 7.9 ± 9 × 10^−2^ (Table A1 in the Appendix A), probably due to the NH_4_^+^ accumulation (Table 3). However, organic acid consumption may also contribute to the effect on the pH.

Moreover, ammonia (measured as free ammonium), an inhibitor of the anaerobic digestion process, showed higher accumulation in the 4:1 experiment, which aligns with the lower biomethane production compared to the 2:1 experiment. At the end of the anaerobic digestion, ammonia concentrations were 1413 ± 11 mg/L and 1275 ± 29 mg/L for initial and spent biomass substrates, respectively, in the 4:1 setup (See Table A1). However, the 2:1 anaerobic digestion experiment resulted in significantly lower final ammonium values: 681 ± 35 mg/L and 656 ± 22 mg/L for initial and spent biomass, respectively (See Table A1). Reductions of up to 50% in biomethane production have been reported in the literature with ammonium concentrations between 1700–1800 mg/L [46], values close to those obtained in this study with an inoculum-to-substrate ratio of 4:1. Thus, ammonium accumulation led to the inhibition of the anaerobic digestion process, resulting in lower biomethane yields using the ratio 4:1. However, in the 2:1 experiments, inhibition did not occur as quickly due to the lower accumulation of ammonia. Additionally, ammonium accumulation was higher when using initial biomass as substrate compared to spent biomass, which aligns with the lower cumulative biomethane production yields observed in Figure 5.

The C/N ratios of the digestates (sludge and sludge + substrate) were analyzed at the end of the anaerobic digestion (day 35 and 30 for 4:1 and 2:1 experiments, respectively). The C/N ratios measured in the digestion at a 4:1 ratio were lower (6.6 ± 0.2 and 6.6 ± 0.3 for initial and spent biomass, respectively) compared to those found in the digestate from the 2:1 anaerobic digestions (10.8 ± 0.2 and 9.2 ± 0.3 for initial and spent biomass, respectively). The low C/N ratios in the 4:1 experiment led to a higher accumulation of ammonium (~5-fold increase). However, in the 2:1 experimental set, ammonium accumulation from day 1 to day 30 was lower: +2.3 for spent biomass and +2.6 for initial biomass, in line with the higher C/N ratios compared to the 4:1 experiment.

**Figure A2:**
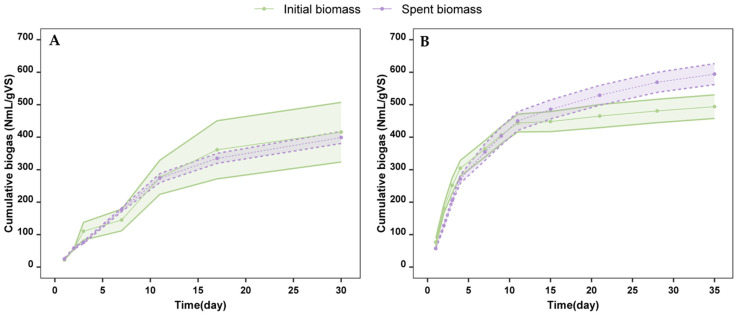
Cumulative total biogas production of initial biomass (green) and spent biomass (purple) of *A. flos-aquae* using inoculum-to-substrate ratios of 4:1 (**A**) and 2:1 (**B**). Initial biomass vs. spent biomass. Normal conditions (T = 273.15 K, P = 101,325 kPa).

Apart from cumulative biomethane production, biogas quality was measured (Figure 5). The experiments with a 4:1 inoculum-to-substrate ratio not only produced low amounts of biomethane but also resulted in low-quality biogas, reaching a maximum of 30.3 ± 1.3% with spent biomass on day 30. The initial biomass at this ratio offered even lower values on day 30 (19.4 ± 0.8%). Moreover, normalized total biogas production was calculated for both inoculum-to-substrate ratios (Figure A2 in Appendix A). In the 4:1 experiment, total biogas production was 416 ± 92 NmL/gVS for the initial biomass and 399 ± 18 NmL/gVS for the spent biomass. These values are relatively high due to the high CO_2_ content of the biogas. No significant differences were observed in biogas production between the two types of substrates.

In contrast, anaerobic digestion at a 2:1 inoculum-to-substrate ratio resulted in higher-quality biogas. The highest biogas quality was obtained with the spent biomass substrate (62.6 ± 0.3%) on day 35, with a total biogas production of 594 ± 32 NmL/gVS. In comparison, the initial biomass produced biogas with a slightly lower quality (58.1 ± 1%) and a total production of 494 ± 36 NmL/gVS. Significant differences in biogas production between the two substrates were observed from day 21 to day 35.

Additionally, the total nitrogen and total phosphate of the soluble digestate were analyzed (see Table A1) to assess its potential as a fertilizer. The nitrogen and phosphate contents increased in all the experiments except for those reactions performed with an inoculum-to-substrate ratio of 2:1 with the initial biomass. Final N:P ratios were 0.4 for initial and spent biomass, when using the inoculum-to-substrate ratio of 4:1, while N:P ratios employing an inoculum-to-substrate ratio of 2:1 were 1.3 and 0.7 for initial and spent biomass, respectively. The ratio 1.3 was close to that recommended (1.7) in one research that assessed the best N:P ratio in fertilizer for obtaining higher wheat yields [47].

### 2.3. Life-Cycle Assessment

**Figure 6:**
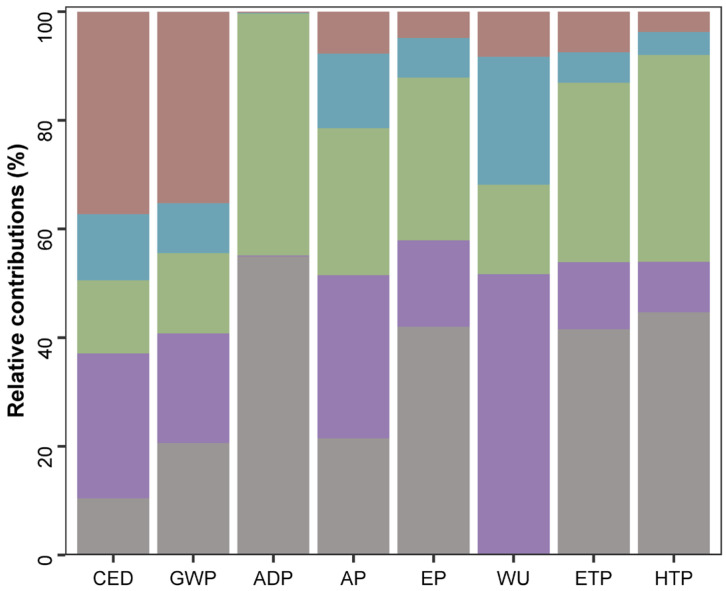
Relative contributions per stage for *A. flos-aquae* biorefinery analyzed by CML 2001 method. Cumulative energy demand (CED), global warming potential (GWP), depletion (ADP), acidification potential (AP), eutrophication potential (EP), water use (WU), eco-toxicity potential (ETP), and human toxicity potential (HTP). *A. flos-aquae* cultivation (gray), harvesting (purple), extraction (green), separation (blue), and extract processing (brown).

The environmental impacts produced in the different stages of the best biorefinery (2:1 inoculum-to-sustrate ratio) are shown in Table 4 and the relative contributions to environmental impact are represented in Figure 6.

*A. flos-aquae* cultivation (stage 1) should contain a culture medium with a mineral source of sodium nitrate and sodium phosphate. The production of these compounds had the highest impact on abiotic depletion (ADP) (54.9%), as shown in Figure 6. The use of those compounds leads to a high contribution in eutrophication potential (EP) by 42.0%, impacting in the environmental indicators of human toxicity potential (HTP) and eco-toxicity potential (ETP) by 44.7% and 41.6%, respectively. This stage had a relatively high contribution to GWP (20.2%) especially due to the production and application of mentioned nutrients, which release substantial amounts of CO_2_ and other greenhouse gases. On the other hand, biomass cultivation had a negligible impact on water use (WU) (0.16%), as water outputs from other stages (centrifugation and freeze drying) were reused. The CED for this stage was also low (10.5%) compared to the other stages, except for the anaerobic digestion stage (stage 6), whose impact was negligible. Cultivation primarily involves supplying light, nutrients, and CO_2_, and maintaining optimal growth conditions, which can often be achieved with minimal energy inputs.

Biomass harvesting (stage 2) after *A. flos-aquae* cultivation contributed highly to WU, accounting for 51.5%. This is due to the water use requirements to produce the equipment and the energy to operate it, like in the separation stage (stage 4), which contributed 23.5% to WU. Consequently, CED was high (26.7%) for biomass harvesting, which also had an impact on GWP. Thus, stage 2 significantly influences the GWP (20.1%), primarily due to the energy-intensive nature of harvesting and the production of materials. AP is high in this stage (30.1%) due to the high energy consumption and emissions, mainly. The EP contribution of this stage was 15.9% due to the presence of soluble compounds recovered in water after centrifugation. However, since this output is reused in subsequent cultivation cycles, it did not result in an important impact on ETP (12.3%) or HTP (9.3%).

The phycocyanin extraction stage (stage 3) required the addition of mineral salts (PBS buffer) to maintain pH conditions for phycocyanin stability, as well as electricity. The production of the PBS buffer, which includes phosphate, mainly responsible for the high contributions found for ADP (44.6%) and the contribution for AP (27.0%). In addition, PBS manufacturing leads to high contributions to EP (30%), ETP (33%), and HTP (38%). WU indicator was 16.4%. Stage 3 involved several steps, such as freeze–thaw cycles and ultrasound-assisted extraction, which required energy. Thus, the CED contribution was 13.4%. This energy consumption contributed to GWP (14.8%) because it involved the use of electricity. The primary emissions were related to the energy used, rather than the extraction process itself. The efficiency of the extraction process meant that less energy was required compared to other stages, such as harvesting (stage 2) and drying (stage 5).

The separation of the supernatant (phycocyanin extract) and spent biomass through centrifugation (stage 4) together with the anaerobic digestion (stage 6) were the steps with the least environmental impact on the overall biorefinery scheme. It should be noted that biogas may require purification depending on its final use, which could introduce an additional step with a high environmental impact, particularly considering that the biogas purity was 62.6% (Figure 5). Digestate can be used to enrich fertilizers due to the N:P ratio. Given the relevance of GWP as a key indicator in environmental assessments, due to its direct link to climate change, it is particularly noteworthy that stages 4 and 6 contributed minimally and negligibly, respectively. In the case of the separation stage (9.2%), this can be attributed to the simplicity and efficiency of the centrifugation process, which involves a single mechanical step with relatively low energy consumption. The negligible contribution of the anaerobic digestion stage to GWP (0.03%) is due to its ability to capture methane, a potent greenhouse gas, and use it as a renewable energy source. The biogas produced reduce reliance on fossil fuels, while the process also converts residual biomass into valuable bioproducts such as fertilizers and biogas.

Finally, in the phycocyanin extract processing stage (stage 5), the CED was found to be the highest (37.2%) in the LCA due to energy-intensive steps such as evaporation and freeze-drying. This leads to a higher GWP (35.2%) since high energy consumption is associated with higher carbon emissions. On the other hand, this step showed low contributions for the rest of the environmental indicators.

As climate change becomes an increasingly critical global issue, GWP serves as a key indicator of its impact. Therefore, it is essential to evaluate GWP values globally in LCA and compare our results with those reported by other researchers in similar biorefineries. The GWP of the whole biorefinery was 3.1 × 10^5^ kg CO_2_ eq (corresponding to 31 per kg of dry phycocyanin extract) (Table 4). This biorefinery contributed less to GWP compared to a similar biorefinery reported by Cogo Badan and coworkers [48]. In their study, GWP values ranged from 3.08 × 10^8^ to 1.36 × 10^8^ kg CO_2_ eq per kg of phycocyanin produced. The difference can be attributed to factors that differ in our study, such as continuous operation for 304 days/year, Nutsche filtration, and sonication during extraction, compared to their use of batch operation for 365 days/year, dynamic cross-filtration, and pulsed electric fields for extraction. Papadaki et al. [49] used *L. platensis* in a biorefinery with a similar extraction method (sonication and buffer). Their study emitted 1.18 × 10^3^ kg CO_2_ and 3.29 × 10^3^ kg CO_2_ eq per kg of phycocyanin, higher than in our study. This difference may stem from their use of energy-intensive cyanobacterium paste drying, which was avoided in our biorefinery. A recent biorefinery for phycocyanin and by-products (small proteins and amino acids) reported GWP contributions of 694–798 kg CO_2_ eq per kg of phycocyanin [50]. Although it contributed less to GWP than the ones mentioned earlier, the biorefinery in the present study is more sustainable, releasing even less CO_2_, likely due to avoiding drying steps and reducing overall process steps, eliminating additional centrifugation.

The LCA conducted in this study can guide future improvements in the biorefinery for *A. flos-aquae* phycocyanin and biogas production. To reduce environmental impact and better reflect real processes, the following actions should be considered: (1) Minimize cumulative energy demand, for example, by using the produced biogas to power energy-intensive equipment, such as photobioreactors or centrifuges. (2) Optimize culture media composition to reduce mineral salt usage. (3) Perform real industrial experiments to determine extraction yields and optimize operational time. (4) Explore alternatives to high-energy equipment (e.g., freeze dryers and sonicators). (5) Consider cleaning, maintenance, and logistics in future assessments.

## 3. Materials and Methods

### 3.1. Cyanobacterium

*A. flos-aquae* biomass was supplied by Cianoalgae (Gipuzkoa, Spain). The concentration of proteins, carbohydrates, lipids, and ash and elemental analysis (N, C, H, S, and O) of *A. flos-aquae* were determined using the methods described in our previous work [27].

### 3.2. Phycocyanin Extraction

Four freeze–thaw cycles were performed to break down the cell walls of *A. flos-aquae*. Briefly, 500 mg of cyanobacterial biomass was mixed with 28 mL of potassium dihydrogen phosphate/di-sodium hydrogen phosphate (PBS) (Scharlab, Barcelona, Spain) for 20 min. at room temperature at 300 rpm. The mixture was frozen using liquid nitrogen and thawed using a water bath at 25 °C. Phycocyanin extraction from *A. flos-aquae* was carried out following the guidelines described by Sánchez-Laso et al. [51], with some modifications. After cell walls had been broken to allow the solubilization of intracellular compounds, the mixture was submitted to ultrasound-assisted extraction (Elmasonic P ultrasound, Elma Schmidbauer GmbH, Singen, Germany) at a frequency of 37 kHz, 80% amplitude, for 25 min. The initial temperature was set at 23 °C, which increased to 32 °C. The mixture was mixed in a vortex device (IKA-Werke GmbH, Staufen, Germany) for 1 min. and centrifugated using a 5910 Eppendorf centrifuge (Hamburg, Germany) at 10,000 rpm for 10 min. The supernatant (phycocyanin rich extract) was separated from the pellet (spent biomass) and filtered through 0.45 µm nylon filters. Phycocyanin extract and residual biomass were stored at −20 °C for analysis.

Phycocyanin (PC) was quantified using spectrophotometry (Cary 500 UV-Vis-NIR spectrophotometer Varian, Inc., Palo Alto, CA, USA) with the Bennet and Bogorad [52] algorithm adapted by another research study [51] as follows:

(1) $$PC=\left(\frac{A_{615}-0.474A_{652}}{5.34}\right)\left(\frac{V\;(mL)}{m\;(g)}\right)$$

where *A*_615_ and *A*_652_ are the absorbances at 615 nm and 652 nm, respectively, and *V* is the volume of extract and *m* the grams of the initial biomass employed in the extractions. Measurements to calculate the phycocyanin content can be found in Appendix A (Table A2).

The concentrations of proteins, carbohydrates, lipids, and ash of aqueous extracts were determined using the methods described in our previous work [27].

#### 3.2.1. Analysis of the Phycocyanin Extracts

*A. flos-aquae* aqueous extracts were analyzed to assess its antioxidant and in vitro cytotoxic effect on two cancer cell lines.

##### Antioxidant Power

ABTS^•+^ assay [53] was used to assess the antioxidant potential of the extracts produced in this work. A total of 2.45 mM of potassium persulfate solution (≥98% purity) from Glentham Life Sciences (Corsham, UK) and 7 mM of 2,2′-azino-bis (3-ethylbenzothiazoline-6-sulfonic acid) diammonium salt 2 (98% purity) from Thermo Fisher Scientific (Waltham, MA, USA) were prepared in MiliQ water, combined in a 1:1 (*v*/*v*) ratio, and left in the dark for 16 h. The absorbance of the stock solution was then adjusted to 0.73 ± 0.03 (734 nm) by diluting it with MiliQ water. After mixing 30 µL of the sample with 600 µL of the stock solution for 10 s, the mixture was left at room temperature in the dark for 7 min.

L(+)-ascorbic acid (Scharlab, Barcelona, Spain) and C-phycocyanin (TargetMol, Boston, MA, USA) standards were prepared in a concentration from 0 to 90 µg/mL and 0 to 3000 µg/mL, respectively, and tested in the ABTS assay to measure its potential to scavenge ABTS^•+^ radicals. ABTS^•+^ scavenging capacity was measured with the following equation:

(2) $${ABTS}^{•+}\;scavenging\;capacity\;(\%)=\left(\left(A_{734B}-A_{734S}\right)/A_{734B}\right)\;100$$

where *A*_734B_ and *A*_734S_ are the absorbances at 734 nm, for the control (MiliQ water) and the sample, respectively.

Antioxidant capacity was also calculated in terms of µg and millimoles of vitamin C (ascorbic acid) per milliliter of extract and per gram of dry extract (VCEAC), respectively, using the first-order algorithms extracted from the vitamin C standard curve. Additionally, scavenging potential was expressed in terms of µg of C-phycocyanin equivalents per mL of extract (C-PC Eq). The concentration of each standard to reach an ABTS^•+^ radicals scavenging of at least 50% (IC_50_) was quantified.

##### Cytotoxicity in Two Cancer Cell Lines

The antitumoral potential of *A. flos-aquae* extract was assessed in two cancer cell lines, supplied by Merck (Darmstadt, Germany): human brain glioma cells (U-118-MG. ATCC-HTB-15) and breast cancer cells (HCC1806. ATCC-CRL-2335), through flow cytometry, a service offered by the Cell Culture Service of the Faculty of Medicine (Universidad de Alcalá, Madrid, Spain). U-118-MG cells were cultured in Dulbecco’s phosphate-buffered saline solution and HCC1806 cells in RPMI-1640 medium, both with 10% fetal bovine serum and 10% antibiotic antifungal solution (100×), all reagents from Merck (Darmstadt, Germany). A total of 150,000 cells per well were cultured and were left for 48 h. After that time, the cells were treated with *A. flos-aquae* extracts at 0.5, 0.75, and 1 mg/mL.

After 48 h of incubation with the aqueous extracts at different concentrations, flow cytometry was carried out. Briefly, the cells were washed with 2 mL of PBS, and trypsin from Merck (Darmstadt, Germany) was used to lift the cells. Then, 2 mL of the culture medium was added to stop the action of trypsin, and the cells were transferred to cytometric tubes. Centrifugation was performed at 15,000 rpm for 7 min. A cellular pellet was suspended in 0.5 mL of PBS and propidium iodide (≥94% purity), supplied by Merck (Darmstadt, Germany), was added. Flow cytometer (MACSQuant^®^ Analyzer 10, Miltenyi, Bergisch Gladbach, Germany) was employed to assess cell mortality. Dot plots were acquired with size versus complexity and size versus B3 (propidium iodide detector). Cells that captured propidium iodide were non-viable.

### 3.3. Biogas Production from the Spent Biomass

After phycocyanin extraction, spent biomass was used to test its potential to produce biomethane in biochemical methane potential (BMP) assays. The sludge (inoculum) used for anaerobic digestion was collected from an anaerobic digester of a wastewater plant in Spain in June 2023. Control experiments containing only the sludge were performed, and the results of biomethane production were subtracted from the experiment’s results. Moreover, the initial biomass of *A. flos-aquae* was also tested for comparison purposes. Two different inoculum-to-substrate ratios were tested: 2:1 and 4:1. Spent biomass was characterized in terms of C/N ratio, total proteins, carbohydrates lipids, and ash content using the methods described in an earlier study [27]. Moisture, volatile solids (VS), and total solids (TS) were measured according to the guidelines of previous work [54]. The VS. content of the inoculum (sludge) was 71.6 ± 1.5% and inoculum-to-substrate ratios were adjusted to 2:1 and 4:1 in terms of vs. content. The moisture content of the substrate (spent biomass) was 80 ± 0.9%. MiliQ water was added to a total volume of 67 mL.

BMP tests were conducted as described in previous work [27,54]. Anaerobic digestion reactions were carried out in 100 mL glass bottles (non-stirred) containing the sludge, the substrate (spent cyanobacterial biomass or intact cyanobacterial biomass), and water, in mesophilic conditions (37 °C). Pressure was measured the first day and monitored using a Pressure Sensor 400 and Vernier Graphical Analysis software version 5.3.1 (Vernier Software & Technology, Beaverton, OR, USA). Biogas samples were taken and analyzed by gas chromatography (GC-4000A, East & West Analytical Instruments, Inc., Beijing, China). After sample collection, bottles were fluxed with nitrogen to create anaerobic conditions. Pressure measurements were performed daily, and biogas was collected and measured when differences among the atmospheric pressure and pressure inside the bottles were found. Experiments performed at an inoculum-to-substrate ratio of 4:1 were carried out for 30 days and a 2:1 inoculum-to-substrate ratio for 35 days.

Digestate was centrifuged at 10,000 rpm for 10 min and then filtered (0.45 µm nylon filters) to obtain the soluble part of the digestate, which was analyzed in terms of chemical oxygen demand (COD), free ammonia (NH_4_^+^), N, and P using COD, NH_4_^+^, N, and P Spectroquant^®^ kits, respectively, supplied by Merck (Darmstadt, Germany).

### 3.4. Statistical Analysis

Three replicates were performed in each experimental section. Normal distribution was first checked using the Shapiro–Wilk test (*p*-value > 0.05). When the data did not follow a normal distribution, they were transformed with different algorithms, depending on the skewness. Levenne’s test (*p*-value > 0.05) was used to check the variance homogeneity. Parametric one-way ANOVA tests were performed to check significant differences between means and Tukey’s HSD (honestly significant difference) post hoc tests were performed to compare the data pairs. Statistical analyses were performed using R software version 4.3.3.

### 3.5. Life Cycle Assessment

**Figure 7:**
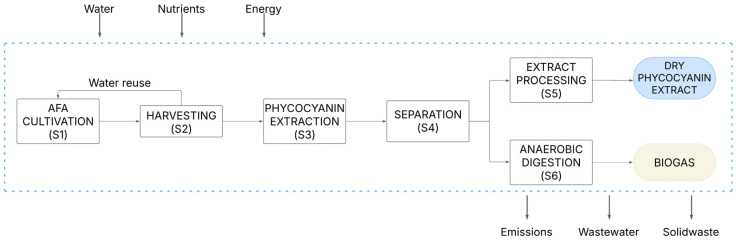
System boundaries of *A. flos-aquae* biorefinery for phycocyanin and biogas production.

LCA was performed to analyze the environmental impacts of the biorefinery to produce dry phycocyanin extract for the cosmetic, food, or nutraceutical sectors. The established functional unit (FU) was 10 tons of phycocyanin per year. The LCA is based on a cradle-to-gate approach: from raw biomass to final product (Figure 7). The logistics, transportation, and maintenance of the equipment were not considered in this approach. Spent biomass was valorized through anaerobic digestion to produce biogas under the concept of circular economy. Consumed energy in the biorefinery scheme can be reduced by using the final biogas to feed the system, starting with the *A. flos-aquae* cultivation phase.

The biorefinery process is composed of 6 stages: *A. flos-aquae* cultivation in a closed photobioreactor (S1), biomass harvesting through centrifugation (S2), cell disruption using a cycle of freeze–thawing and the phycocyanin extraction using ultrasounds (S3), a separation stage obtaining the extract and the residual biomass (S4), the extract processing in which it is filtered with a Nutsche filtration system, dewatered by means of a flash evaporator, and frozen-dried (S5), and the final stage that is anaerobic digestion to produce biogas (S6).

**Figure A3:**
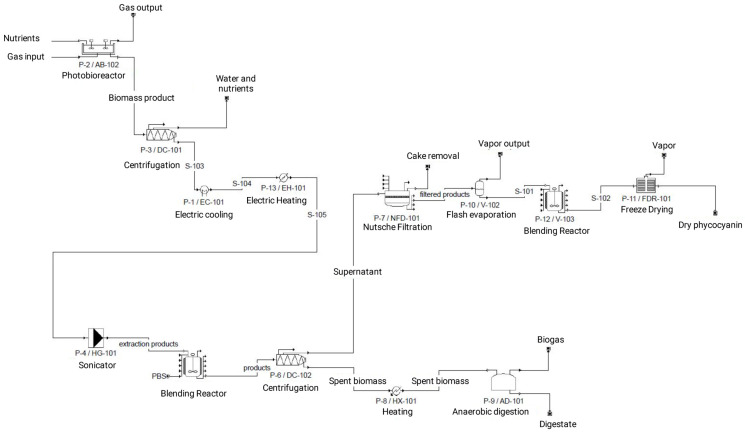
Simulation of the biorefinery scheme. Water is recirculated.

The simulation of the overall process, including all the stages, was performed with SuperPro Designer 9.5 (Figure A3), obtaining the life cycle inventory (Table 5). This inventory data were used to calculate the environmental impacts of the phycocyanin dry extract production, using CML 2001 through Gabi 6.0 software.

## 4. Conclusions

This study successfully developed a biorefinery for *A. flos-aquae*, yielding valuable phycocyanin and biogas from spent biomass. The extract demonstrated significant antioxidant activity (77% at 2.4 mg/mL), though only a fraction (22%) was attributable to pure C-phycocyanin, emphasizing the importance of utilizing the whole extract for optimal antioxidant effects. Notably, the extract exhibited potent cytotoxicity against HCC1806 breast cancer cells (80.9% mortality at 1 mg/mL), exceeding the observed cytotoxicity in U-118 MG glioma cells (47.5%). Interestingly, C-phycocyanin alone showed significant cytotoxicity only against glioma cells, highlighting the complex interplay of bioactive compounds in the phycocyanin extract. Furthermore, the spent biomass proved to be an excellent substrate for biogas production, delivering high yield and quality. Finally, an LCA study of the scaled-up process was carried out to identify its most important environmental hotspots, highlighting the need to reduce the use of fertilizers for biomass cultivation, as well as to minimize the energy consumption in the extraction process. This research underscores the potential of *A. flos-aquae* as a versatile resource for both high-value products and renewable energy, emphasizing the need for the strategic selection of raw materials to maximize desired bioactivities.

## Figures and Tables

**Table 1:** Characterization of *A. flos-aquae* intact biomass (IB), aqueous extract (AE), spent biomass (SB), and biomolecule recovery in the aqueous extract and spent biomass.

Biomolecule	Biochemical Characterization (%, Dry Basis)			Biomolecule Recovery (%)	
	IB	AE	SB	AE	SB
Proteins	65.7 ± 3.2	73.8 ± 4.7	77.6 ± 1.4	46.7 ± 2.8	30.5 ± 0.6
Carbohydrates	19.0 ± 0.3	20.8 ± 1.3	9.6 ± 0.6	45.6 ± 2.8	13.0 ± 0.8
Lipids	5.9 ± 0.1	1.8 ± 0.1	3.7 ± 2.7	12.6 ± 0.5	15.9 ± 2.5
Ashes	5.5 ± 0.0	3.6 ± 0.1	9.2 ± 0.4	27.0 ± 0.6	43.0 ± 0.3
Total	96.1 ± 3.6	100 ± 6.2	100.1 ± 5.1	43.2 ± 0.3	26.9 ± 0.1

**Table 2:** Antioxidant capacity of *A. flos-aquae* aqueous extracts. Results obtained from ABTS^•+^ assay.

Antioxidant Indicator	Value
ABTS^•+^ radicals scavenging (%)	77.0 ± 0.7% (2.4 mg/mL)
VCEAC (µg/mL)	235 ± 2.1
VCEAC (mmol/g dry extract)	0.17 ± 1 × 10^−3^
C-PC Eq (µg/mL)	9971 ± 90

**Table 3:** Measured variables to test anaerobic digestion performance.

	ISR ^1^	COD ^2^	NH_4_^+ 3^	C/N ^4^
Initial biomass	4:1	+2.4	+4.8	6.6 ± 0.2
Spent biomass	4:1	+1.9	+5.2	6.6 ± 0.3
Sludge	4:1	-	-	7.9 ± 4.5 × 10^−2^
Initial biomass	2:1	−6.3	+2.6	10.8 ± 0.2
Spent biomass	2:1	−1.8	+2.3	9.2 ± 0.3
Sludge	2:1	-	-	11.9 ± 0.3

^1^ ISR: inoculum-to-substrate ratio; ^2^ chemical oxygen demand, expressed as fold change; ^3^ ammonium, expressed as fold change; and ^4^ carbon-to-nitrogen ratios in sludge + substrate (final day).

**Table 4:** Environmental impacts of the different stages of the biorefinery. Cumulative energy demand (CED), global warming potential (GWP), abiotic depletion (ADP), acidification potential (AP), eutrophication potential (EP), water use (WU), eco-toxicity potential (ETP), and human toxicity potential (HTP).

	Biomass Cultivation (S1)	Harvesting (S2)	Extraction (S3)	Separation (S4)	Extract Processing (S5)	Anaerobic Digestion (S6)	Total
CED (MJ)	5.5 × 10^5^	1.4 × 10^6^	7.1 × 10^5^	6.4 × 10^5^	1.9 × 10^6^	1.5 × 10^3^	5.3 × 10^6^
GWP (kg CO_2_ eq.)	6.3 × 10^4^	6.1 × 10^4^	4.5 × 10^4^	2.8 × 10^4^	1.1 × 10^5^	8.3 × 10	3.1 × 10^5^
ADP (kg Sb eq.)	2.1 × 10	6.8 × 10^−3^	1.7 × 10	3.1 × 10^−3^	9.3 × 10^−3^	7.0 × 10^−6^	3.9 × 10
AP (kg SO_2_ eq.)	4.2 × 10^2^	5.8 × 10^2^	5.3 × 10^2^	2.7 × 10^2^	1.5 × 10^2^	7.5 × 10^−2^	1.9 × 10^3^
EP (kg Phosphate eq.)	3.0 × 10^2^	1.1 × 10^2^	2.1 × 10^2^	5.2 × 10	3.5 × 10	1.9 × 10^−2^	7.1 × 10^2^
WU (m^3^ world equiv.)	6.6 × 10^4^	2.1 × 10^7^	6.6 × 10^6^	9.5 × 10^6^	3.4 × 10^6^	1.1 × 10^3^	4.0 × 10^7^
ETP (kg DCB eq.)	6.2 × 10^2^	1.9 × 10^2^	5.0 × 10^2^	8.5 × 10	1.1 × 10^2^	7.6 × 10^−2^	1.5 × 10^3^
HTP (kg DCB eq.)	1.9 × 10^5^	2.3 × 10^4^	9.3 × 10^4^	1.0 × 10^4^	9.2 × 10^3^	5.6 × 10	2.4 × 10^5^

**Table 5:** Life Cycle Inventory. Data referred to FU.

**S1:** ** *A. flos-aquae* cultivation**	Closed photobioreactor	Nutrients (NaH_2_PO_4_ + NaNO_3_)	Input	52.6 × 10^3^	kg
		Water (reused + new input)	Input	10.2 × 10^7^	kg
		Gas (air)	Input	51.3 × 10^6^	kg
		Gas (CO_2_)	Input	19.6 × 10^3^	kg
		Gas (air)	Output	51.3 × 10^6^	kg
		Gas (CO_2_)	Output	2.8 × 10^3^	kg
		Biomass	Output	57.8 × 10^3^	kg
**S2: Harvesting**	Centrifuge	Biomass	Input/output	57.8 × 10^3^	kg
		Electricity	Input	4.3 × 10^5^	MJ
**S3: Phycocyanin extraction**	Electric cooler + electric heater +Sonicator	Biomass	Input	57.8 × 10^3^	kg
		Extraction products	Output	57.8 × 10^3^	kg
	Blending reactor	Extraction products	Input/output	57.8 × 10^3^	kg
		PBS (disodium phosphate + KH_2_PO_4_)	Input	9.3 × 10^3^	kg
		Electricity (total S3)	Input	1.4 × 10^5^	MJ
**S4: Separation**	Centrifuge	Extraction products	Input	57.8 × 10^3^	kg
		PBS (disodium phosphate + KH_2_PO_4_)	Input	9.3 × 10^3^	kg
		Electricity	Input	2.0 × 10^5^	MJ
		Supernatant	Output	55.1 × 10^3^	kg
		Spent biomass	Output	2.8 × 10^4^	kg
**S5: Extract processing**	Nutsche filtration + Flash evaporator + Blending reactor + Freeze dryer	Supernatant	Input	55.1 × 10^3^	kg
		Heat	Input	1.3 × 10^6^	MJ
		Electricity (total S5)	Input	4.3 × 10^4^	MJ
		Dry phycocyanin extract	Output	10	tons
**S6: Anaerobic digestion**	Heating + Anaerobic digestion	Spent biomass	Input	2.8 × 10^4^	kg
		Heat	Input	1.1 × 10^3^	MJ
		Biogas	Output	3.5	tons
		CO_2_	Output	2.4	tons
		CH_4_	Output	1.1	tons
		Digestate	Output	24	tons

## Data Availability

Data upon request to interested researchers.

## Abbreviations

The following abbreviations are used in this manuscript:

ABTS^•+^	2,2′-azino-bis(3-ethylbenzothiazoline-6-sulfonic acid
ADP	Abiotic depletion
AP	Acidification potential
C-PC Eq	C-phycocyanin equivalents
CED	Cumulative energy demand
COD	Chemical oxygen demand
EP	Eutrophication potential
ETP	Eco-toxicity potential
FU	Functional unit
GWP	Global warming potential
HCC1806	Breast cancer cell line
HTP	Human toxicity potential
ISR	Inoculum-to-substrate ratio
LCA	Life cycle assessment
PC	Phycocyanin
TS	Total solids
U-118 MG	Brain glioma cell line
VCEAC	Vitamin C equivalents
VS	Volatile solids
WU	Water use

## Appendix A

**Table A1:** Characterization of soluble fraction of the mixtures on day 0 of experimentation and on the last day. * Day *n*: Last day of BMP tests; 30 days for 4:1 I:S ratio and 35 days for 2:1 I:S ratio.

		4:1		2:1	
Parameter	Substrate	Day 0	Day *n* *	Day 0	Day *n* *
NH_4_^+^ (mg/L)	Initial biomass	297 ± 16	1413 ± 11	263 ± 6	681 ± 35
	Spent biomass	243.3 ± 4	1275 ± 29	287 ± 2	656 ± 22
COD (mg/L)	Initial biomass	2652 ± 183	6194 ± 83	2013 ± 91	320 ± 13
	Spent biomass	2329 ± 82	4498 ± 216	472 ± 1	265 ± 3
Total nitrogen (mg/mL)	Initial biomass	156 ± 2	546 ± 28	195 ± 0	289 ± 0
	Spent biomass	113 ± 0	531 ± 27	152 ± 6	240 ± 20
Total phosphate (mg/mL)	Initial biomass	727 ± 11	1400 ± 118	266 ± 3	228 ± 6
	Spent biomass	605 ± 24	1312 ± 70	176 ± 4	334 ± 12
pH	Initial biomass	6.3 ± 2 × 10^−2^	5.3 ± 2 × 10^−2^	6.9 ± 3 × 10^−2^	7.9 ± 5 × 10^−2^
	Spent biomass	6.4 ± 0	5.3 ± 2 × 10^−2^	7.0 ± 4 × 10^−2^	7.9 ± 9 × 10^−2^

**Table A2:** Spectroscopic data obtained from *A. flos-aquae* aqueous extracts used to determine phycocyanin content.

ID	Initial Biomass (mg)	Solvent Volume (mL)	*A*_615_	*A*_652_	Dilution	PC (mg/g)
17	501.8	28	1.172	0.733	0.2	43.08
18	505.9	28	1.138	0.709	0.2	41.60
19	506.0	28	1.148	0.716	0.2	41.92
20	503.4	28	1.116	0.694	0.2	41.02
21	509.7	28	1.156	0.721	0.2	41.90
22	502.0	28	1.155	0.722	0.2	42.46
23	500.2	28	1.123	0.700	0.2	41.47
24	505.1	28	1.134	0.706	0.2	41.52
